# Supplementary material for: Metabolic Biomarkers Affecting Cell Proliferation and Prognosis in Polycythemia Vera
Source: Cancers (Basel). 2022 Oct 7;14(19):4913. doi: 10.3390/cancers14194913 (PMC9563618; doi:10.3390/cancers14194913)
Supplement: Supplementary file 1 [file cancers-14-04913-s001.zip › cancers-1912812-supplementary.pdf]

cancers

MDPI

*Supplementary Files*

# Metabolic Biomarkers Affecting Cell Proliferation and Prognosis in Polycythemia Vera

**Table S1.** Clinical characteristics of the PV patients.

| Descriptive Parameters | PV (n=32) | JAK2 mutated (n=24) | JAK2 unmutated (n=8) | *P* |
|---|---|---|---|---|
| Gender[n(%), female/male] | 9 / 23 | 9 / 15 | 0 / 8 | 0.07 |
| Age[year, M(range)] | 54.5 (21~77) | 64 (40~77) | 38.5 (21~62) | 0.001 |
| WBC[$\times 10^9$/L, M(range)] | 7.595 (4.45, 20.95) | 9.35 (4.45, 20.95) | 6.98 (4.59, 9.1) | 0.151 |
| HGB[g/L, M(range)] | 190 (167, 223) | 191 (167, 216) | 182 (177, 223) | 0.429 |
| PLT[$\times 10^9$/L, M(range)] | 302 (111,752) | 366 (111,752) | 225.5 (157,394) | 0.006 |
| HCT[%, M(range)] | 55.8 (49.8, 69) | 57.2 (50.6, 69) | 52.8 (49.8, 60.6) | 0.044 |
| JAK2 mutation burden [%, M(range)] | - | 65.8 (10.4, 93.3) | - | - |
| JAK2 mutation[n(%)] | - | 24（75） | - | - |
| JAK2 V617F mutation[n(%)] | - | 22 (68.75) | - | - |
| JAK2 exon12 mutation[n(%)] | - | 2 (6.25) | - | - |
| CALR mutation[n(%)] | 0（0） | - | - | - |
| MPL mutation[n(%)] | 0（0） | - | - | - |
| Diabetes [n(%)] | 2 (6.25) | 1 (4.17) | 1 (12.5) | 0.444 |

PV: polycythemia vera; WBC: white blood cell count; HGB: hemoglobin; PLT: platelet count; NEUT: neutrophil count; HCT: hematocrit.

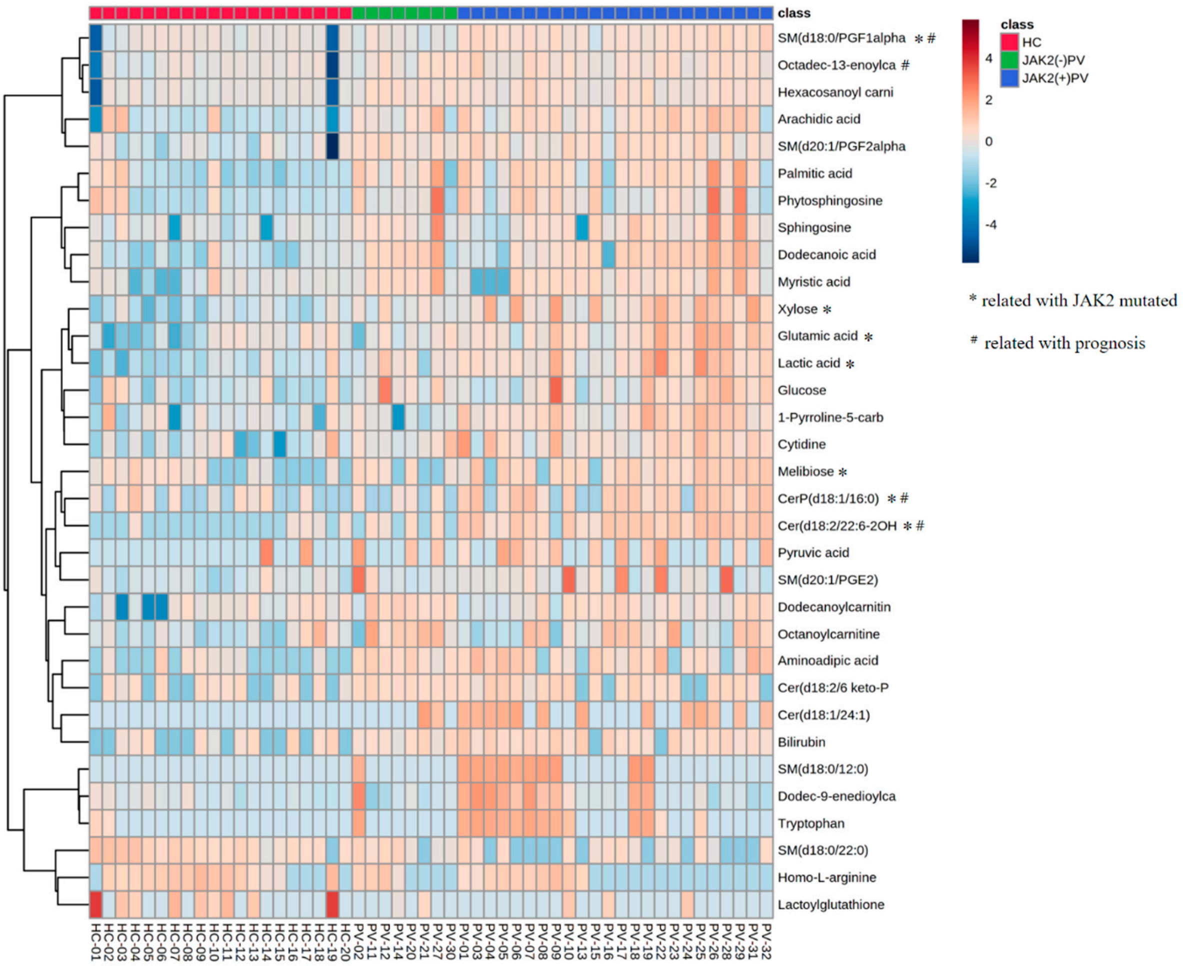


**Figure S1.** Hierarchical clustering heatmap of potential biomarkers for PV patients.

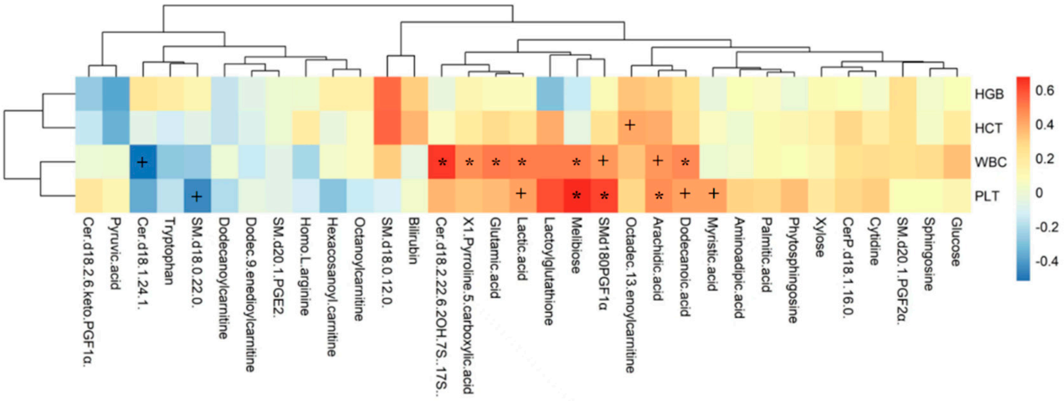


**Figure S2.** Heatmap of correlation between differential metabolites in PV patients and WBC, HGB, PLT and HCT at diagnosis. Spearman's correlation test, $^{+}$ $P < 0.05$, $^{*}$ $P < 0.01$.
